# Acceleration of postoperative recovery with brief intraoperative vagal nerve stimulation mediated *via* the autonomic mechanism

**DOI:** 10.3389/fnins.2023.1188781

**Published:** 2023-06-19

**Authors:** Alimujiang Maisiyiti, Ming Tian, Jiande D. Z. Chen

**Affiliations:** ^1^Department of Minimally Invasive Surgery, Hernias and Abdominal Wall Surgery, People's Hospital of Xinjiang Uygur Autonomous Region, Urumqi, China; ^2^Department of Surgery, Dongzhimen Hospital, Beijing University of Chinese Medicine, Beijing, China; ^3^Division of Gastroenterology and Hepatology, University of Michigan School of Medicine, Ann Arbor, MI, United States

**Keywords:** vagal nerve stimulation, postoperative recovery, neuromodulation, gastrointestinal motility, inflammatory cytokines

## Abstract

**Introduction:**

Postoperative recovery is largely dependent on the restoration of gastrointestinal motility. The aim of this study was to investigate the effects and mechanisms of intraoperative vagus nerve stimulation (iVNS) on postoperative recovery from abdominal surgery in rats.

**Methods:**

The Nissen fundoplication surgery was performed on two groups of rats: sham-iVNS group and iVNS group (VNS was performed during surgery). Animal’s behavior, eating, drinking and feces’ conditions were monitored at specific postoperative days. Gastric slow waves (GSWs) and electrocardiogram (ECG) were recorded; blood samples were collected for the assessment of inflammatory cytokines.

**Results:**

(1) iVNS shortened initiate times to water and food intake (*p* = 0.004) and *increased the* number of fecal pellets (*p* < 0.05, vs. sham-iVNS) and the percentage of water content in fecal pellets (*p* < 0.05). (2) iVNS improved gastric pace-making activity at 6 h after surgery reflected as a higher percentage of normal slow waves (*p* = 0.015, vs. sham-iVNS). (3) iVNS suppressed inflammatory cytokines at 24 h after surgery compared to sham-iVNS (TNF-α: *p* = 0.001; IL-1β: *p* = 0.037; IL-6: *p* = 0.002). (4) iVNS increased vagal tone compared to sham-iVNS group at 6 h and 24 h after the surgery (*p* < 0.05). Increased vagal tone was correlated with a faster postoperative recovery to start water and food intake.

**Conclusion:**

Brief iVNS accelerates postoperative recovery by ameliorating postoperative animal behaviors, improving gastrointestinal motility and inhibiting inflammatory cytokines mediated *via* the enhanced vagal tone.

## Introduction

The Healthcare Cost and Utilization Project estimated 7 million surgical discharges in 2014 with a cost of $158 billion. Postoperative recovery is the main influencing factor in hospital stay (HCUP National Inpatient Sample (NIS). Healthcare Cost and Utilization Project (HCUP), 2016); promoting early recovery after surgery is critical in reducing healthcare costs and improving patients’ satisfaction. Accordingly, enhanced recovery after surgery (ERAS) was introduced by Henrik Kehlet in 1990s (Bardram et al., 1995; Kehlet, 1997). ERAS is a multidisciplinary program aiming at reducing the surgical stress response and organ dysfunctions. The main components of ERAS include preoperative education, anesthesia, prevention of intraoperative hypothermia, perioperative fluid management, minimally invasive techniques, postoperative pain management, dietary regulation and early mobilization. However, despite all of these efforts, an undesirable portion of surgical patients still suffer from some postoperative complaints such as pain, nausea, vomiting and postoperative ileus (Kuntz et al., 1998; Wilmore and Kehlet, 2001; Anderson et al., 2003; Kehlet and Wilmore, 2008). In addition to surgery and trauma, postoperative opioid analgesics also contribute to the incidence of postoperative ileus.

Reduced gastrointestinal motility, post-operative pain and inflammation are considered main contributing factors for the postoperative recovery following abdominal surgeries. Autonomic nervous system dysfunction, gastrointestinal hormone disruptions and inflammatory responses induced by surgical injury are main causes of these postoperative complications (Tracey, 2002).

The vagus nerve is known to play an important role in controlling heart rate, gastrointestinal motility and secretion, pancreatic endocrine and exocrine secretion, hepatic glucose production, and other visceral functions. In addition, the vagus nerve is a major constituent of inflammatory reflex that controls innate immune responses and inflammation (Tracey, 2002, 2009; Andersson and Tracey, 2011). Vagus nerve stimulation (VNS) has been shown to exert anti-inflammatory effects in inflammatory bowel diseases (IBD; Bonaz et al., 2016, 2017; John et al., 2017). VNS has been reported to improve inflammation, gastrointestinal motility and to reduce postoperative pain mediated *via* the autonomic mechanisms in both animals and humans (Wang et al., 2010; Yuan and Silberstein, 2016; Jin et al., 2017).

Recently, intraoperative VNS (iVNS) was introduced for treating postoperative ileus (Wong et al., 2017; Murakami et al., 2018). A previous study in our lab showed that iVNS prevented the delay of gastric emptying and intestinal tissue damages in a rodent model of postoperative ileus (O’Neil et al., 1990; Murakami et al., 2018). However, it was an acute and temporary study lasting only for 3 h. To further investigate the role of iVNS in postoperative recovery, the Nissen fundoplication procedure was selected and outcome measures were monitored for 72 h after surgery.

The aims of this study were to investigate postoperative recovery by assessing animal behaviors, postoperative recovery of gastric functions assessed by the gastric pace-making activity and to explore mechanisms involving autonomic functions and inflammatory cytokines.

## Materials and methods

### Preparations of animals

Eighteen eight-week-old adult male Sprague–Dawley rats (300–350 g) were purchased from Charles River Laboratories International, Inc., Wilmington, MA. The rats were housed in regular cages before surgery in a temperature-controlled environment at 22°C, humidity 40%, on a 12-h light, 12-h dark cycle. Animals had free access to a standard rat laboratory diet and water (Murakami et al., 2018). However, they were overnight fasted with *ad libitum* access to water before surgery. The animal experiment was carried out in accordance with the National Institutes of Health Guide for the Care and Use of Laboratory Animals and the experimental protocol was approved by the Johns Hopkins University Animal Care and Use Committee, Baltimore City, MD, United States. The authors were working at the Johns Hopkins University at the time of the study.

### Study protocol

**Figure 1:**
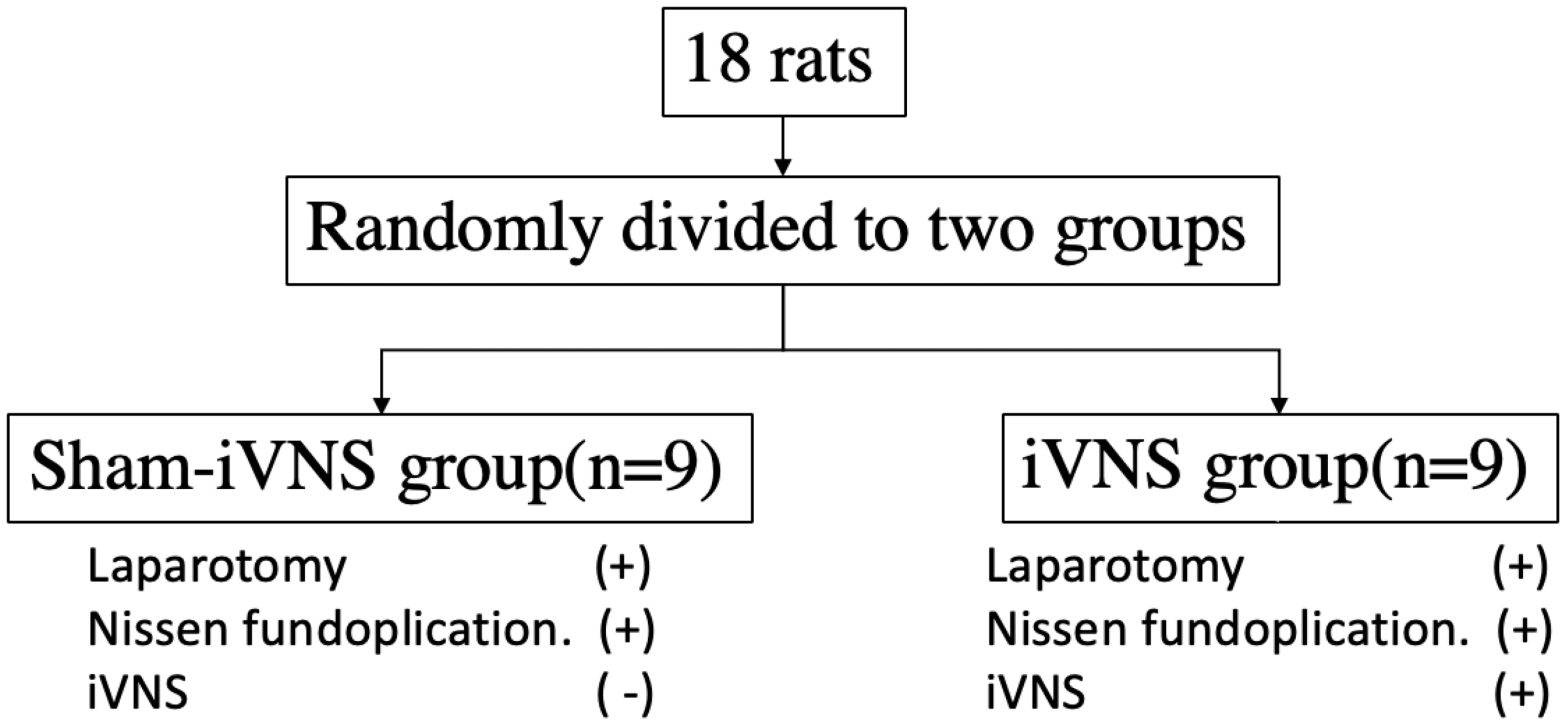
Randomization of groups.

**Figure 2:**
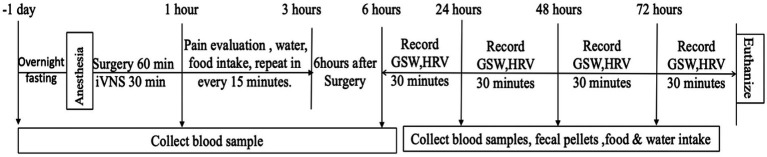
Study protocol.

The rats were randomly divided into sham-iVNS group (*n* = 9) and iVNS group (*n* = 9; Figure 1). The Nissen fundoplication surgery was performed on both sham-iVNS group and iVNS group. iVNS/sham-iVNS was performed during the first 30-min of the Nissen fundoplication procedure in the corresponding group. Animal behaviors, eating, drinking and feces were monitored at specific postoperative periods. Gastric slow waves (GSWs) and electrocardiogram (ECG) were recorded (Wang et al., 2010; Jin et al., 2017); Rat Grimace Scale (RGS) was used to assess pain (Sotocinal et al., 2011); Blood samples were collected for the assessment of inflammatory cytokines at specific postoperative times. The detailed experimental protocol is shown in Figure 2.

### Surgical procedures

The rat was overnight fasted with water *ad libitum*, cephalexin (15 mg/kg, S.C.) and Buprenorphine (0.05 mg/kg, S.C.) were administered 1 h before surgery. Anesthesia was induced by 2% isoflurane and oxygen flow of 1–2 L/min in a chamber. The rat was placed on a heating pad, and anesthesia was maintained *via* a breathing mask attached to the rat. The abdominal wall of the rat was depilated from xiphoid to groin using an electric hair clipper and prepared with an iodine solution followed with 70% isopropanol. All surgical procedures were done aseptically (Sotocinal et al., 2011; Xu et al., 2011; Jin et al., 2017; Murakami et al., 2018).

#### Nissen fundoplication

A midline abdominal incision of 5–7 cm length was made. The gastro-hepatic ligament was identified and sectioned. The distal esophagus was gently grasped with small forceps and the retro esophageal membrane was dissected by blunt mobilizations. Through this hole, the gastric fundus was wrapped 360° around the distal esophagus and the gastric fundus was sutured to the anterior gastric wall using two stitches with a 4–0 absorbable suture. The stomach was then placed back to its original location; after completion of all procedures including iVNS (detailed below), the abdominal muscle was closed using a continuous stitch of a 4–0 absorbable suture and sterile saline (20 mL/kg) was administered subcutaneously to prevent dehydration after surgery.

#### Electrode placement

Electrodes (cardiac pacing wires, A&E Medical, Farmingdale, NJ) were implanted at different locations for iVNS, ECG recording and GSWs recording under anesthesia in all animals. A pair of electrodes was placed circumferentially around the posterior (dorsal) vagus nerve below the diaphragm for iVNS. Three electrodes were implanted for ECG recording on the chest and abdominal wall subcutaneously (Jin et al., 2017). One pair of electrodes was sutured on the gastric serosa of the antrum with an interval of 1 cm. The connecting wires for ECG and GSWs recordings were tunneled subcutaneously and externalized at the back of the neck. The external wires were fixed with a silk suture on the skin (Jin et al., 2017; Murakami et al., 2018).

### Intraoperative VNS and sham-iVNS

iVNS was performed using a universal pulse generator (Model DS8000, World Precision Instruments, Sarasota, FL, United States) during the first 30 min of the surgery using following parameters: 5 Hz, 0.5 ms, 2.2 mA, 10s on, 90s off, previously optimized to treat intestinal inflammation (Murakami et al., 2018). In the sham-iVNS group, electrodes were implanted exactly same as the iVNS group but no current was delivered to the vagal nerve.

### Observation of postoperative animal behaviors

Observations of post-operative animal behaviors were mainly focused on the first post-surgical time to water intake and food intake (counted starting from wake-up of the animal from anesthesia), the amount of 24-h water intake and food intake, the number of 24-h pellets on postoperative days 1 to 3. The first post-surgical time to water intake and food intake was assessed from a video recording (also used for pain evaluation) that was initiated immediately after placing the rat in wired-bottom cage after surgery (Murakami et al., 2018).

The water and food were weighed at the beginning and ending of every 24-h to calculate the amount of water intake and food intake. Cages were changed every 24 h and fecal pellets were collected. Weight of fecal pellets were measured at collection and at the time when pellets were dried out (kept in an exhaust hood for 3 days) to calculate the water content in feces (Jin et al., 2017).

### Evaluation of postoperative pain

After the surgery, the rat was housed individually in a wire bottom cage. A digital camera (D600, Canon, Japan) was placed outside the acrylic glass walls of the cage to get clear head shots and record behavioral aspects. The digital movies were taken for 5 min each at 15, 30, 60, 90, 120, 150, and 180 min after the surgery. The Rat Grimace Scale (RGS) for pain evaluation was scored by two evaluator who were totally blinded to this study (Sotocinal et al., 2011). The RGS consisted of four facial “action units” (orbital tightening, nose/cheek appearance, ear and whisker positions) which were scored using still images by the observer. The observer assigned a value of 0, 1, or 2 for each of the 4 RGS action units. The final RGS score was the average score across the 4 action units (Sotocinal et al., 2011).

### Assessment of autonomic functions

The autonomic function was assessed by the spectral analysis of the heart rate variability (HRV) signal derived from the ECG (Murakami et al., 2018). The ECG signal was recorded for 30 min using a special amplifier (model 2,283, Fti Universal Fetrode Amplifier, UFI, Morro Bay, CA, United States) at different postoperative time periods. Previously validated software (Xu et al., 2011) was used to derive the HRV signal from the original ECG recording by identifying R waves, interpolating R-R interval data at 100 Hz, and finally down-sampling the interpolated HRV data to 8 Hz for spectral analysis. Two frequency bands in the power spectrum of the HRV were calculated as follows: (1) high frequency (HF): 0.8–4.0 Hz, reflecting purely vagal activity and (2) low-frequency (LF): 0.3–0.8 Hz, reflecting mainly sympathetic activity. The ratio of LF/HF reflected the sympathovagal balance (Xu et al., 2011; Jin et al., 2017; Murakami et al., 2018).

### Recording and analysis of GSWs

GSWs were recorded for 30 min at 6 h, 24 h, and 72 h after the surgery using a Biopac system (EOG 100A; Biopac Systems, Santa Barbara, CA; Murakami et al., 2018). Parameters of the GSW were derived from spectral analysis using previously validated software (Liu et al., 2004a,b). The power at the dominant frequency (DF) in power spectrum was defined as the dominant power (DP). The percentages of normal slow waves, bradygastria and tackygastria were calculated according to the percentage of time during which regular 4–6 cycle/min (cpm) waves, waves of <4 cpm and waves of >6 cycle/min (cpm) were presented, respectively (Liu et al., 2004a,b).

### Assessment of inflammatory cytokines

Cytokines levels in plasma were measured for assessment of postoperative inflammation. Blood samples were collected one day before the surgery as baseline and recollected at 1 h, 6 h, 24 h, and 72 h after the surgery, and were centrifuged at 3000 × g for 10 min at 4°C. TNF-α, IL-1β, and IL-6 levels in plasma were assessed using corresponding commercial ELISA kits (Abcam, Cambridge, MA) according to the protocols provided by the manufacturer. The absorbance rate was read at 450 nm. The concentrations of the samples were calculated according to the standard curve.

### Statistical analysis

The data are presented as mean ± SD. Student’s t-test was used for the comparison between the two groups and one-way ANOVA was used to compare the difference in any of the measurements among different post-surgical points, followed with Tukey’s test using SPSS. Statistical significance was set at *p* < 0.05.

## Results

### Effects of iVNS on postoperative animal behaviors

**Figure 3:**
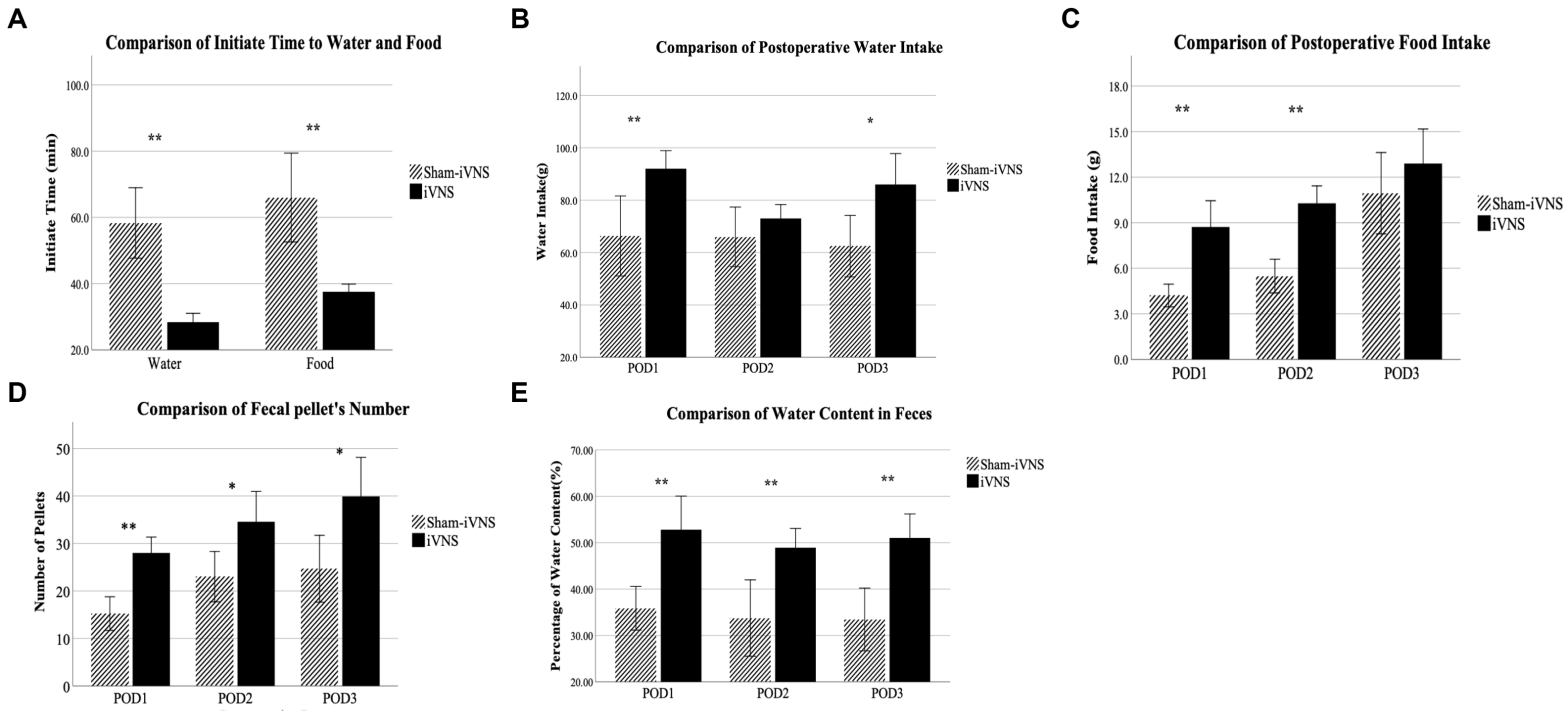
Observation of postoperative animal behaviors.

iVNS accelerated postoperative recovery by shortening the first post-surgical times of water and food intake, increasing the amounts of water and food intake, and increasing the number of fecal pellets and percentage of water content in fecal pellets. iVNS substantially reduced not only the first post-surgical time of water intake (28.4 ± 3.9 min vs 58.3 ± 15.9 min，p = 0.001) but also the first post-surgical time of food intake (37.6 ± 3.5 min. vs 66.0 ± 20.1 min, *p* = 0.003) compared to sham-iVNS (no stimulation) (Figure 3A). iVNS increased the amount of water intake (92.0 ± 10.3 vs. 66.3 ± 22.9, *p* = 0.011, vs. sham-iVNS) and food intake (8.7 ± 2.6 vs 4.2 ± 1.1 g, *p* = 0.001, vs. sham-iVNS) on postoperative day one (POD1); the effect on food intake remained significant on POD2 (10.3 ± 1.7 vs 5.5 ± 1.7 g, *p* = 0.001), whereas the effect on water intake was not significant on POD2 but significant on POD3 (86.0 ± 17.7 vs 62.4 ± 17.6 g, *p* = 0.012; Figures 3B,C).

Compared with sham-iVNS, iVNS increased the number of pellets on POD1-3 (POD1: *p* = 0.001; POD2: *p* < 0.013; POD3: *p* < 0.014; Figure 3D). On POD1-3, the water content in feces was 52.8, 48.9 and 51% in the iVNS group compared with 35.9, 33.8 and 33.4% in the sham-iVNS group (*p* = 0.01, *p* = 0.005, *p* = 0.001), respectively (Figure 3E). No diarrhea was observed in any of the rats. No correlation was noted between the food intake and number of fecal pellets (*r* = 0.256, *p* = 0.306) on POD3.

### Effects of iVNS on postoperative pain

**Figure 4:**
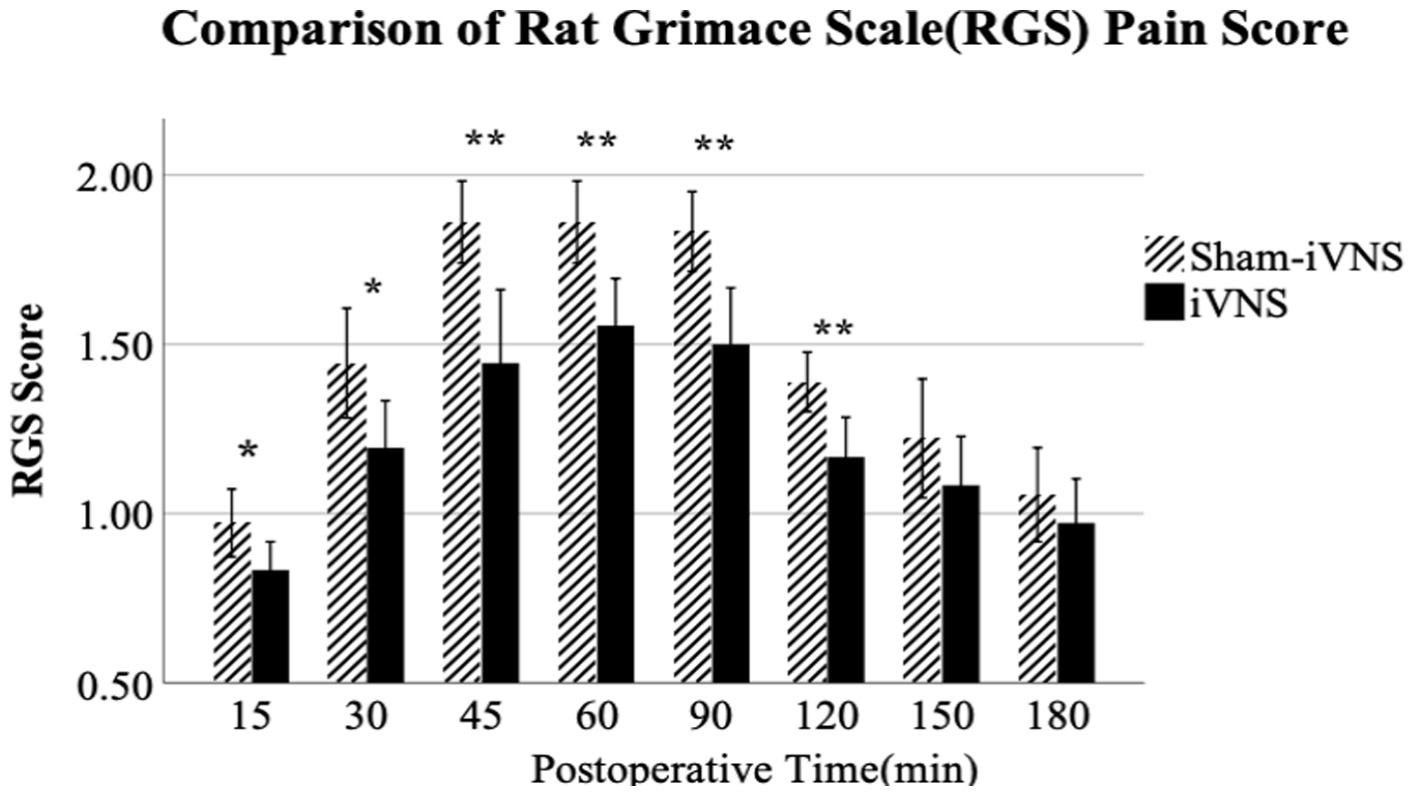
Evaluation of post operative pain.

iVNS reduced postoperative pain assessed by the analysis of RGSs. There were significant differences in the RGSs at 15 and 30 min after surgery between iVNS and sham-iVNS (15 min: 0.83 ± 0.13 vs. 0.97 ± 0.15, *p* = 0.049; 30 min: 1.19 ± 0.21 vs. 1.44 ± 0.24, *p* = 0.032). These differences became more significant at 45 min to 120 min (45 min: 1.44 ± 0.13 vs. 1.86 ± 0.18, *p* = 0.004; 60 min: 1.56 ± 0.33 vs. 1.86 ± 0.18, *p* = 0.004; 90 min: 1.50 ± 0.25 vs. 1.83 ± 0.18, *p* = 0.005; 120 min: 1.17 ± 0.18 vs. 1.39 ± 0.13, *p* = 0.008). However, there were no significant differences between iVNS and sham-iVNS at post-operative time 150 min and 180 min (Figure 4).

### Effects of iVNS on gastric pace-making activity

**Figure 5:**
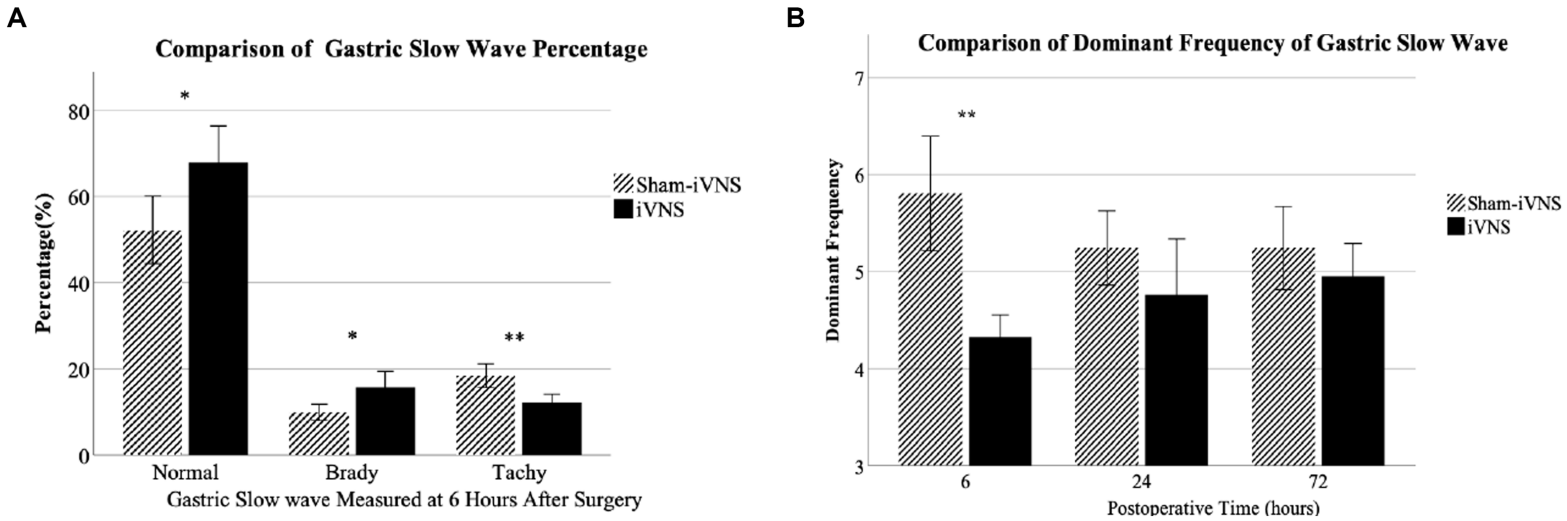
Effects of iVNS on gastric pace-making activity.

iVNS improved gastric pace-making activity at 6 h after surgery: a higher percentage of normal slow waves was observed in the iVNS group compared to the sham-iVNS group (67.9 ± 12.7% vs. 52.2 ± 11.8%, *p* = 0.015, vs. sham-iVNS; Figure 5A). There was a decrease in the percentage of tackygastria and an increase in bradygastria with iVNS at the same time period. However, these differences were no longer significant at 24 h or 72 h after the surgery.

The DF in the iVNS group was significantly lower than that in the sham-iVNS group 6 h after surgery (4.33 ± 0.34 vs. 5.81 ± 0.89, *p* = 0.001). At 24 h and 72 h after the surgery, there were no significant differences in DF between iVNS and sham-iVNS (Figure 5B). There were no significant differences in dominant power at any post-operative periods between the two groups.

### Mechanisms involving inflammatory cytokines

**Figure 6:**
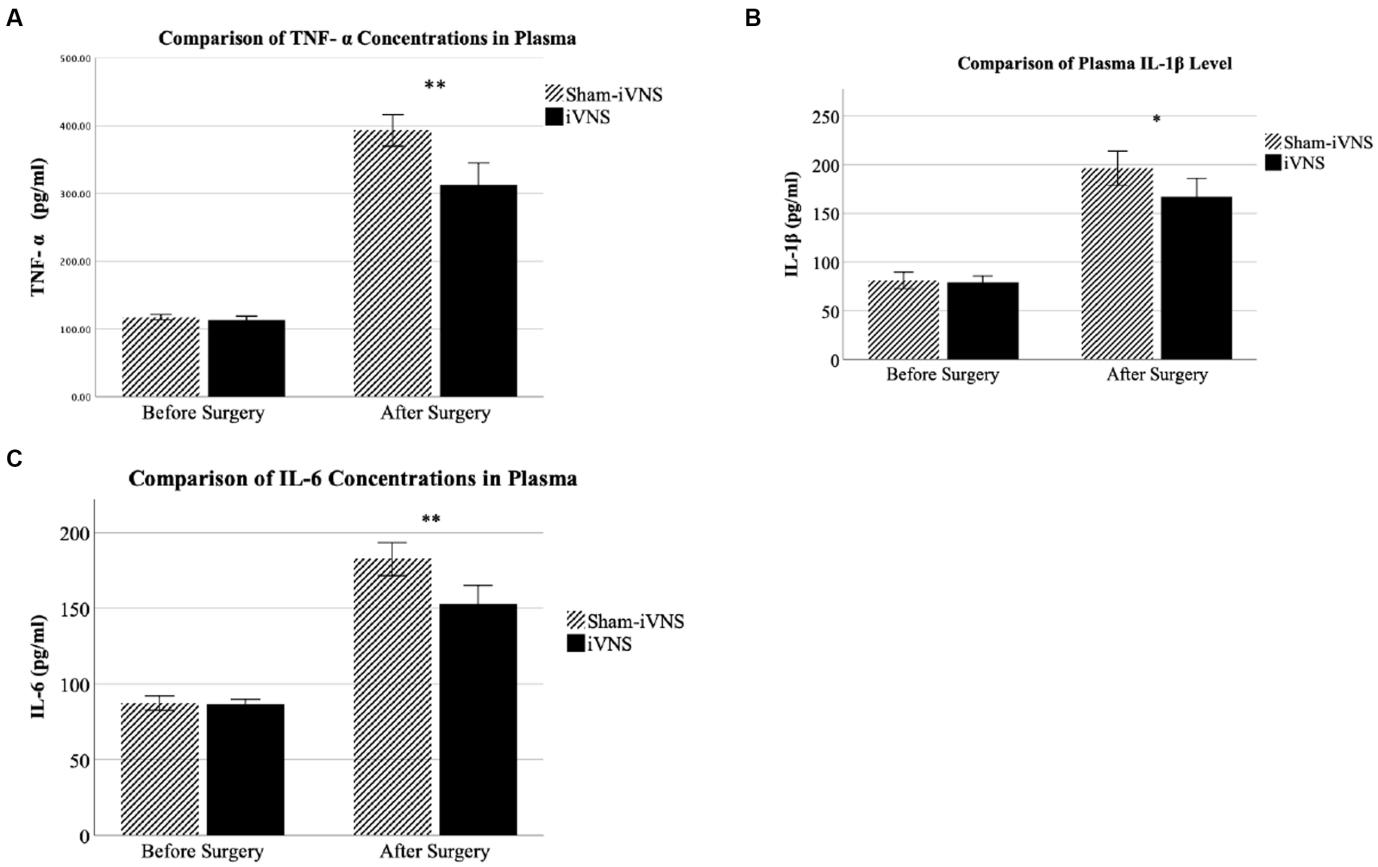
Mechanisms involving inflammatory cytokines.

The concentrations of TNF-α, IL-1β, and IL-6 in plasma were increased after the surgery in both sham-iVNS and iVNS group, and this increase was reduced in the iVNS group compared to the sham-iVNS group (Table 1; TNF-α: 311.9 ± 48.9 vs. 392.9 ± 34.7, p = 0.001; IL-1β: 167.2 ± 28.4 vs. 196.5 ± 26.3, *p* = 0.037; IL-6: 152.9 ± 18.2 vs. 182.5 ± 16.4, *p* = 0.002; Figures 6A–C).

**Table 1:** TNF-a, IL-1β, and IL-6 levels in plasma.

Cytokines	Sham-iVNS		iVNS	
	Pre-operative	Post-operative	Preoperative	Postoperative
TNF-α (pg/mL)	117.2 ± 5.8	392.9 ± 34.7	112.7 ± 9.3	311.9 ± 48.9*
IL-1β (pg/mL)	81.2 ± 12.7	196.5 ± 26.3	79.2 ± 9.8	167.2 ± 28.4*
IL-6 (pg/mL)	87.3 ± 7.1	182.5 ± 16.4	86.5 ± 4.9	152.9 ± 18.2*

*p < 0.05 vs. Sham-iVNS

### Mechanisms involving autonomic functions

iVNS improved vagal activity that suppressed by the surgical procedure. iVNS increased vagal tone compared to sham-iVNS at 6 h (HF: 0.58 ± 0.12 vs. 0.38 ± 0.09, *p* = 0.001) and 24 h (HF: 0.57 ± 0.08 vs. 0.49 ± 0.05, *p* < 0.017) after the surgery (Figure 6A). Similarly, there was a decrease in LF/HF with iVNS at 6 h and 24 h after the surgery (Figure 6B). These effects were diminished as the vagal tone spontaneously recovered. Most interestingly, the iVNS seemed to be able to prevent the surgery-induced decrease in vagal tone as its value at 6 h was even higher than that at 72 h after the surgery.

**Figure 7:**
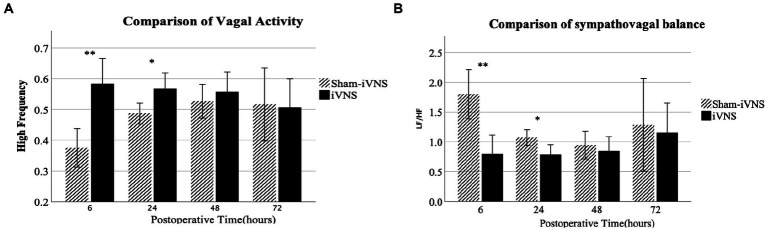
Mechanisms involving autonomic functions.

**Figure 8:**
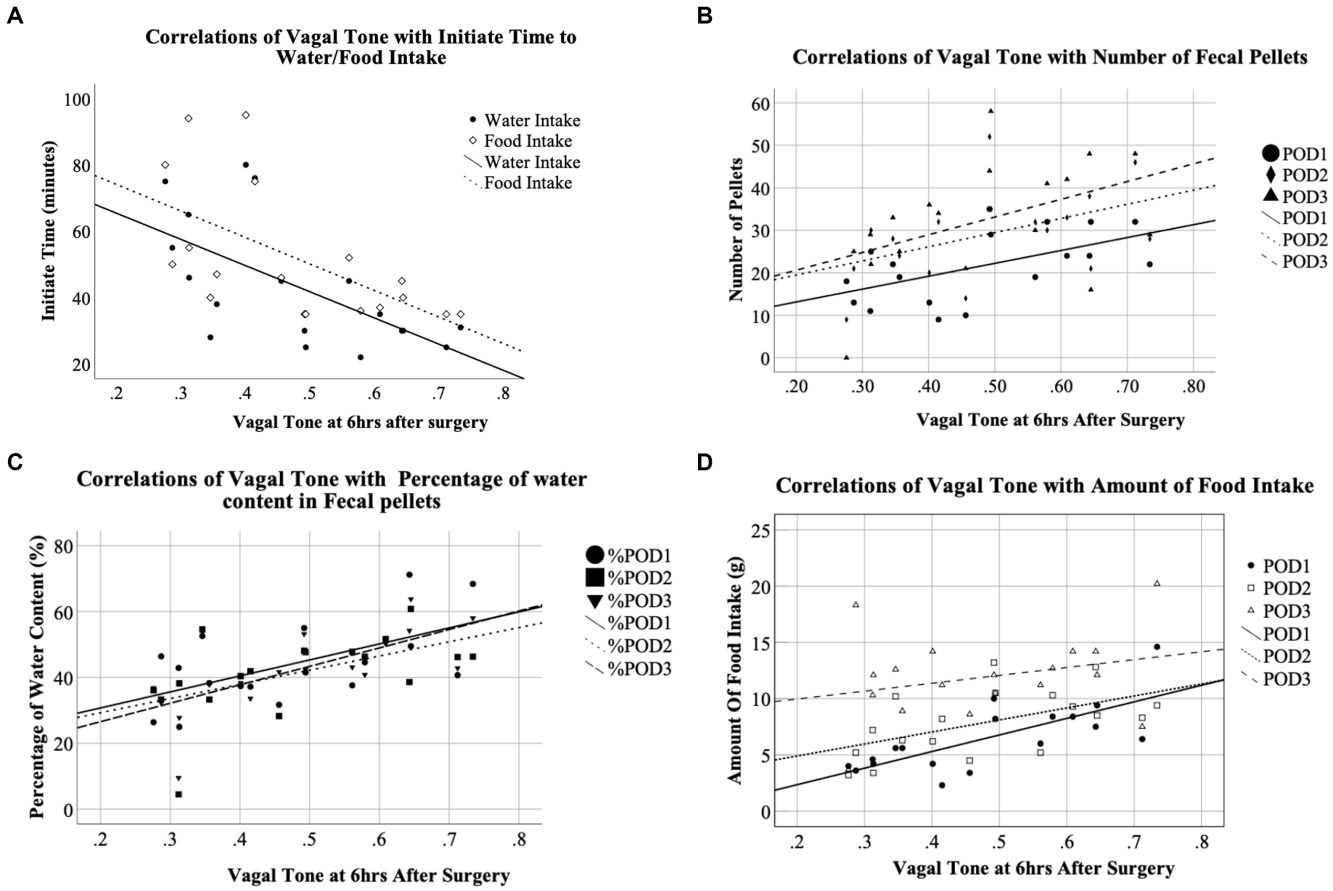
Correlations of autonomic functions with animal behavior.

The increased vagal tone was correlated with a faster postoperative recovery to start water and food intake; the correlation was stronger at earlier postoperative time periods. The vagal tone at 6 h after the surgery was negatively correlated with the first post-surgical time of water (*r* = −0.623, *p* = 0.006) and food intake (*r* = −0.594, *p* = 0.009). The vagal tone at 24 h after the surgery was moderately and negatively correlated with the first post-surgical time of water intake (*r* = −0.511, *p* = 0.03). No significant correlations were found at 48 and 72 h after the surgery (Figures 7, 8).

Increased vagal tone was also correlated with the increased number of fecal pellets; the correlation was stronger at earlier postoperative times. Vagal tone at 6 h after the surgery was positively correlated with the number of fecal pellets on POD1 (*r* = 0.552, *p* < 0.018) and POD2 (*r* = 0.482, *p* < 0.044). Vagal tone at 24 h after the surgery was positively correlated with the number of fecal pellets on POD1 (*r* = 0.572, *p* = 0.013) and POD3 (*r* = 0.477, *p* = 0.045). No significant correlations were found between the vagal tone at 48 or 72 h and the number of fecal pellets.

Interestingly, increased vagal tone was also correlated with the percentage of water content in the fecal pellets. Vagal tone at 6 h after the surgery was positively correlated with the percentage of water content in the fecal pellets on POD1 (*r* = 0.588, *p* < 0.01), POD2 (*r* = 0.528, *p* = 0.024) and POD3 (*r* = 0.669, *p* = 0.002).

Moreover, increased vagal tone was correlated with increased amount of food intake and the correlation was stronger at earlier postoperative times. Vagal tone at 6 h was positively correlated with the amount of food intake at POD1 and POD2 (POD1: *r* = 0.733, *p* = 0.001; POD2: *r* = 0.542, *p* = 0.02). Vagal tone at 24 h was positively correlated with the amount of food intake at POD1 and POD2 (POD1: HF: *r* = 0.506, *p* = 0.032; POD2: *r* = 0.635, *p* < 0.005). No significant correlations were found between food intake and vagal tone at 48 h or 72 h.

Increased vagal tone was correlated with the decreased level of inflammatory cytokines in plasma on the entire postoperative period (HF with TNF-α: *r* = −0.775, *p* = 0.001; HF with IL-1β: *r* = −0.856, *p* = 0.001; HF with IL-6: *r* = −0.888, *p* = 0.001).

## Discussion

In this study, we have demonstrated that brief iVNS accelerated postoperative recovery by ameliorating postoperative animal behaviors, alleviating postoperative pain, improving gastrointestinal motility and inhibiting inflammatory cytokines mediated by enhancing vagal tone. The experimental findings have demonstrated that iVNS (1) shortened the time of the first post-surgical intake of water and food, (2) increased the amount of food intake, (3) increased the number of pellets and the percentage of water content in feces, (4) exerted an analgesic effect on postoperative pain, (5) improved postoperative pace-making activity and (6) exerted anti-inflammatory effect by suppressing TNF-α, IL-1β and IL-6 levels in plasma. Concurrently, iVNS enhanced vagal activity and the enhanced vagal activity was correlated with the improvement of a number of post-surgical behavioral and physiological measurements.

To investigate the potential role of iVNS in accelerating post-surgical recovery, we have chosen the Nissen fundoplication as a surgical procedure and performed a brief 30-min VNS during the surgery. Since gastrointestinal motility is one of the major contributing factors in post-surgical recovery, we assessed the gastric pace-making activity as a surrogate for gastric motility, and the number of fecal pellets and water content in feces as a surrogate for colon motility. Gastric pace-making activity is the basic controlling element of gastric contraction, whereas the increased number of fecal pellets and the increased water content in feces are indicative of enhanced colon transit. The experiment was designed to prove the hypothesis that iVNS enhances vagal efferent activity, and the enhanced vagal efferent activity suppresses post-surgical inflammation and impairment in gastrointestinal motility, leading to an accelerated postsurgical recovery.

In the present study, iVNS significantly decreased not only initiate time to water and food intake, but also significantly increased postoperative water intake and food intake compared to sham-iVNS. The enhancive effect of iVNS on food intake remained significant until 48 h after the surgery. Enhanced recovery after surgery (ERAS) protocols, first established in 1999, involve several postoperative objectives including short recovery time, improved patient satisfaction, early mobilization, pain control and early oral intake (Lam et al., 2019).

There were reports about vagal nerve stimulation improve gastrointestinal motility. Previous studies have reported that abdominal surgery decreased intestinal slow wave frequency and rhythmicity, and IM impaired the recovery of the slow wave frequency and rhythmicity. The VNS prevented these IM-induced delays in slow wave recovery and improved intestinal transit in STZ-induced diabetic rats (Su et al., 2013). Fang et al. reported improvement of gastric emptying and intestinal transit including the contents of the stomach, small bowel cecum, and colon in POI by VNS (Tatewaki et al., 2003; Iwa et al., 2007; Lin et al., 2007; Chen et al., 2008; Su et al., 2013).

In the present study, iVNS improved gastric pace-making activity. There was a decrease in the percentage of tackygastria and an increase in bradygastria with iVNS at the same time period. iVNS improved vagal activity which was suppressed by the surgical procedures. The iVNS significantly increased vagal tone, there was a decrease in LF/HF with iVNS at 6 h and 24 h after the surgery. Most interestingly, the iVNS seemed to be able to prevent the surgery-induced decrease in vagal tone as its value at 6 h was even higher than that at 72 h after the surgery.

Increased vagal tone was correlated with a faster postoperative recovery to start water and food intake and the correlation was stronger at earlier postoperative time periods. Increased vagal tone was also correlated with the increased number of fecal pellets; the correlation was stronger at earlier postoperative times. Interestingly, increased vagal tone was also correlated with the percentage of water content in the fecal pellets. Moderate increase of water content in the fecal pellets is correlated with better relief of constipation. Moreover, increased vagal tone was correlated with increased amount of food intake and the correlation was stronger at earlier postoperative times. These findings seemed to suggest that the iVNS-induced acceleration in postoperative recovery was attributed to the enhancement of vagal activity. This was in agreement with several previous studies in which electrical stimulation performed noninvasively *via* acupuncture points was reported to enhance vagal activity and the enhanced vagal activity was associated with accelerated postoperative recovery (Li et al., 2020; Zhu et al., 2020; Zhang et al., 2021).

### Clinical perspectives/implications

The findings of this study suggest that brief intra-surgical vagal nerve stimulation may lead to an accelerated recovery from the surgical procedure. The abdominal surgical procedure may benefit most from iVNS due to its relatively higher prevalence of postoperative ileus. The surgical procedure with easy access to the vagus nerve is best suited for applying iVNS as no extra procedure would be required. Clinical studies are needed to prove the translatability of the animal findings to humans. However, several previous studies with the use of noninvasive auricular vagal nerve stimulation have demonstrated the promotility effect of vagal nerve stimulation. In one study, transcutaneous cervical vagal nerve stimulation applied a few minutes daily gastric emptying and symptoms of gastroparesis (Gottfried-Blackmore et al., 2020). In another study, daily transcutaneous auricular vagal nerve stimulation improved symptoms and gastric motility functions in patients with functional dyspepsia (Zhu et al., 2021). The similar method of transcutaneous auricular electrical stimulation also improved constipation in patients with irritable bowel syndrome (Yin et al., 2010; Shi et al., 2021). Both of these studies demonstrated improvement in vagal activity with transcutaneous auricular vagal nerve stimulation (Shi et al., 2021; Zhu et al., 2021).

In summary, brief intra-surgical vagal nerve stimulation accelerates postoperative recovery reflected as a shortened time for the first defecation and increased food intake. The accelerative effect might be attributed to improved gastrointestinal motility mediated *via* the enhancement of vagal activity.

## Data availability statement

The original contributions presented in the study are included in the article/supplementary material, further inquiries can be directed to the corresponding author.

## Ethics statement

The animal study was reviewed and approved by Johns Hopkins University Animal Care and Use Committee.

## Author contributions

AM carried out the experiment and wrote the manuscript with support from JC. MT was responsible for animal surgery. JC conceived the original idea and supervised the project. All authors contributed to the article and approved the submitted version.

## Conflict of interest

The authors declare that the research was conducted in the absence of any commercial or financial relationships that could be construed as a potential conflict of interest.

## Publisher’s note

All claims expressed in this article are solely those of the authors and do not necessarily represent those of their affiliated organizations, or those of the publisher, the editors and the reviewers. Any product that may be evaluated in this article, or claim that may be made by its manufacturer, is not guaranteed or endorsed by the publisher.
